# Allocation of Healthcare Professional Resources in Outpatient Specialised Psychiatric Care: Insights from the First Two Years Post-Depression Diagnosis

**DOI:** 10.1192/j.eurpsy.2026.10970

**Published:** 2026-07-31

**Authors:** P. Näätänen, P. Vänni, G. Joffe, J. Ekelund, J. Juntura, T. Ito, O. Isomeri, I. Eriksson, P. Torkki

**Affiliations:** 1 Department of Psychiatry, Helsinki University Hospital; 2 Department of Public Health, University of Helsinki, Helsinki; 3 Nordic Healthcare Group, Espoo, Finland; 4 Johnson & Johnson, High Wycombe, United Kingdom; 5 Johnson & Johnson, Solna, Sweden

## Abstract

**Introduction:**

Appropriate, timely allocation of healthcare professional (HCP) resources in specialised psychiatric care (SPC) is key for delivering value-based healthcare. Healthcare systems are challenged with balancing outpatient and inpatient care, and optimising consultation, assessment, and treatment planning and administration to maximise patient (pt) outcomes (Wallang *et al.* Br J Hosp Med 2018; 79 322–327). Reviewing real-world resource utilisation can identify deviations from optimal care processes and improve pt contact with HCPs, leading to better outcomes.

**Objectives:**

To analyse HCP resource allocation over 2 years (yrs) post-depression diagnosis by outpatient SPC, examining contact frequency, service type, and the effects of age and sex.

**Methods:**

This cohort study leveraged Finnish electronic health records from 2014–2020. All pts with a depression diagnosis recorded by SPC at Helsinki University Hospital (HUS) in 2018 (with no depression diagnosis given by SPC within the previous 4 yrs) were included. All HCP resource use data, including outpatient services with structurally coded service parameters (type and provider), were evaluated.

**Results:**

A total of 4,830 pts were included. The proportion of consultations, assessments and care plans, and contact with medical doctors, was highest in the first 2 months post-diagnosis vs the remaining follow-up period, during which contact with nurses was highest (**Fig A–B**). For pts aged 18–64 yrs, HCP contact was more than twice as frequent in the first month post-diagnosis vs subsequent months (**Fig C**). For pts aged 0–17 yrs, overall HCP contact was ~2 times higher and more consistent over the follow-up than for pts aged 18–64 yrs. This increased care was primarily provided by nurses and psychologists; hence, the proportion of contact with medical doctors was lower for pts aged 0–17 vs 18–64 yrs. Pts aged 0–22 yrs received proportionally more psychotherapeutic interventions vs other age groups. The frequency and diversity of HCP contact was lowest for pts aged >65 yrs. There were no differences in care between sexes in the first month post-diagnosis; however, female pts received more frequent care than male pts during the remaining follow-up period (**Fig D**).

**Image 1:**
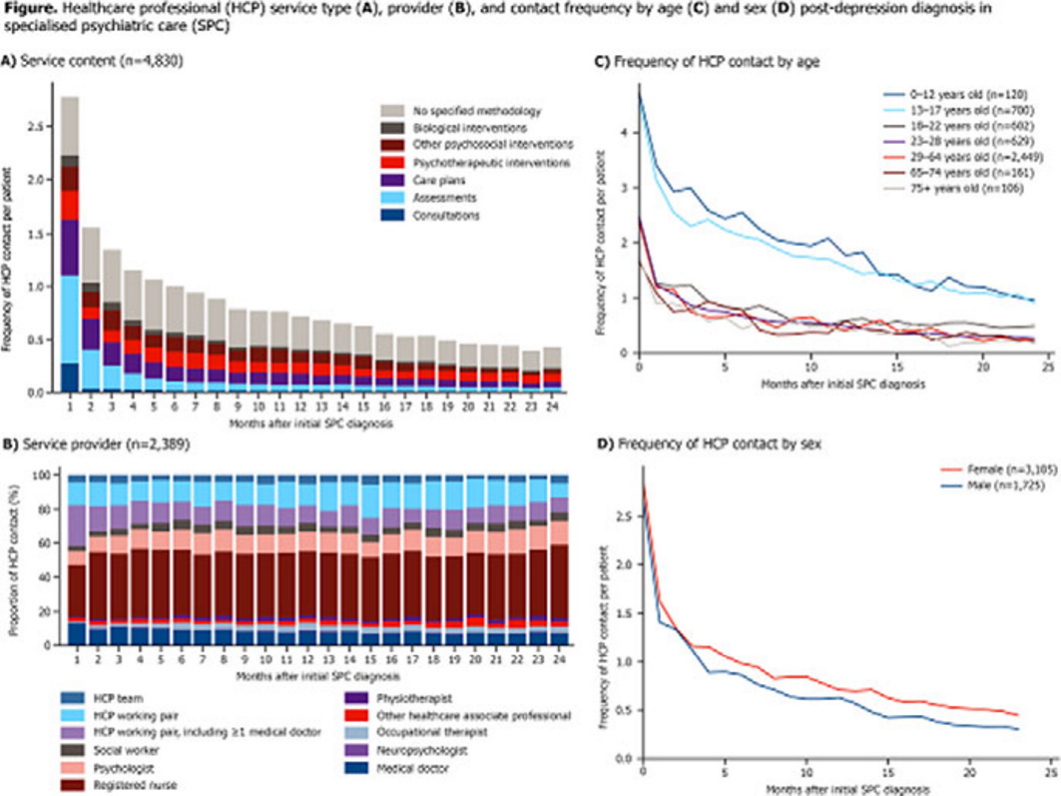
Image 1: Long description.

**Conclusions:**

The findings suggest that the allocation of services to younger pts in Finland is more comprehensive than for older pts, whose care process may be long and diluted. This may be due to limitations or ineffective allocation of resources. Females received more frequent care than males, despite a higher male suicide risk associated with depression, highlighting a need for research into sex disparities. Addressing these issues with efforts in service design and outcomes measurement could improve the effectiveness and equity of depression care in Finland.

**Acknowledgements:**

Funding: HUS, University of Helsinki and J&J; medical writing: Costello Medical.

**Disclosure of Interest:**

P. Näätänen: None Declared, P. Vänni Consultant of: Employer (Nordic Healthcare Group) received a consulting fee for the work., G. Joffe: None Declared, J. Ekelund: None Declared, J. Juntura: None Declared, T. Ito Shareholder of: Holds Johnson & Johnson company stock/stock options, Employee of: Employee of Johnson & Johnson, O. Isomeri Consultant of: Employer (Nordic Healthcare Group) received a consulting fee for the work, I. Eriksson Shareholder of: Holds Johnson & Johnson company stock/stock options, Employee of: Employee of Johnson & Johnson, P. Torkki: None Declared

## Long descriptions

### Image 1: Long description

Panel A is a stacked bar chart titled Service content (n=4,830). The y-axis is Frequency of H C P contact per patient (0 to 2.5) and the x-axis is Months after initial S P C diagnosis (1 to 24). The total frequency starts high at 2.75 in month 1 and decays exponentially to approximately 0.4 by month 24. The stack includes Consultations, Assessments, Care plans, Psychotherapeutic interventions, Other psychosocial interventions, Biological interventions, and No specified methodology. Consultations and Assessments are most frequent in the first 3 months.

Panel B is a 100 percent stacked bar chart titled Service provider (n=2,389). The y-axis is Proportion of H C P contact (0 to 100 percent). The distribution remains relatively stable over 24 months. Key providers include Medical doctor (dark blue at bottom), Psychologist (dark brown), and Registered nurse (light pink). Other categories include H C P team, Social worker, and Physiotherapist.

Panel C is a line graph titled Frequency of H C P contact by age. The y-axis is Frequency of H C P contact per patient (0 to 4) and the x-axis is Months after initial S P C diagnosis (0 to 25). It shows 7 age groups. The 0-12 and 13-17 years old groups show significantly higher contact frequencies (starting at 4.5) compared to adult groups (starting between 1.5 and 2.5). All groups show a sharp decline in the first 5 months.

Panel D is a line graph titled Frequency of H C P contact by sex. The y-axis is Frequency of H C P contact per patient (0 to 2.5). It compares Female (n=3,105) and Male (n=1,725). Both follow a similar downward trend from month 0 to 24, with females maintaining a slightly higher frequency of contact than males from month 5 onwards.

Navigate back to Image 1:.

